# The evaluation of depression prevalence and its association with obesity phenotypes in a community-dwelling aged population

**DOI:** 10.1007/s40520-024-02904-6

**Published:** 2024-12-21

**Authors:** Faezeh Abbasloo, Pouya Ebrahimi, Delaram Ghadimi, Farshad Sharifi, Arian Ayati, Mitra Moodi, Masoumeh Khorashadizadeh, Hosein Fakhrzadeh, Amin Zaki Zadeh, Pedram Ramezani, Reza Pirdehghan, Sara Nooraeen, Ali Moradi, Moloud Payab, Mahbube Ebrahimpur

**Affiliations:** 1 Endocrinology & Metabolism Research Institute, Faculty of Medicine, Tehran University of Medical Sciences, Tehran, Iran; 2 Endocrinology and Metabolism Population Sciences Institute, First Floor, Non-Communicable Diseases Research Center, Tehran University of Medical Sciences, No 10, Jalal-Al-Ahmad Street, North Kargar Avenue, Tehran, 14117-13137 Iran; 3 Faculty of Medicine, Shahid Beheshti University of Medical Sciences, Tehran, Iran; 4 Elderly Health Research Center, Endocrinology and Metabolism Population Sciences Institute, First Floor, Tehran University of Medical Sciences, No 10, Jalal-Al-Ahmad Street, North Kargar Avenue, Tehran, 14117-13137 Iran; 5 Social Determinants of Health Research Center, Birjand University of Medical Sciences, Birjand, Iran; 6 Faculty of Medicine, Ahwaz Jundishapur University of Medical Sciences, Ahwaz, Iran; 7 Health and Wellness Department, George Brown College, Toronto, ON Canada; 8 Department of Psychiatry, University of Pittsburgh, Pittsburgh, PA USA; 9 Center for Translational Medicine, Faculty of Medicine, Semmelweis University, Budapest, Hungary

**Keywords:** Aging, Depression, Metabolic syndrome, Mood disorders, Obesity

## Abstract

**Background:**

Depression is one of the most debilitating mental disorders and a risk factor for many other chronic diseases that are commonly seen in the geriatric population. It has been claimed in previous studies that depression can be associated with obesity in this age group, but there is no common consensus between their results.

**Aim:**

This study aims to evaluate the association between depression metabolic syndrome and obesity phenotypes in community-dwelling older adults living in the East of Iran.

**Method and materials:**

As a part of the Birjand Longitudinal Aging Study, this retrospective cross-sectional study was conducted on participants older than 60. They were categorized based on their body mass index and components of metabolic syndrome into four phenotypes: metabolic non-healthy obese (MNHO), metabolic healthy obese (MHO), metabolic healthy non-obese (MHNO), and metabolic non-healthy non-obese (MNHNO). The relative risk ratio (RRR) of the obesity phenotypes, the severity of depressive symptoms, and the 95% confidence intervals (95% CI) were evaluated by univariate and multinomial logistic regression.

**Results:**

Of 1344 eligible participants, 268 (19.94%) had depression. Moderate, moderate-severe, and severe depression were observed in 179 (13.32%), 67 (4.99%), and 22 (1.64%) participants, respectively. Our findings showed a non-significant increase in the RRR of mild depressive symptoms in MNHO (RRR:1.22, 95% CI 0.56–2.66) and severe symptoms in MNHNO (RRR:1.20, 95% CI 0.02–63.17) females. However, in male participants, the RRR of moderate-severe depressive symptoms only increased non-significantly for the MNHO category (RRR:1.34, 95% CI 0.45–3.98).

**Conclusion:**

We did not observe a meaningful association between depressive symptoms and obesity phenotypes. Also, other than malnutrition or its risk, various severities of depressive symptoms correlate with different sociodemographic and medical risk factors among male and female senior citizens.

## Introduction

Considering the increasing trend of life expectancy due to overall improvement in health care, there has been a surge in the older adult population and the rate of many chronic diseases worldwide, such as depression [1] [2]. Although several mental and psychiatric conditions accompany older adult’s depression. Depression is one of the most common, debilitating, and negatively effective diseases in older adults, with an estimated prevalence of 17% in the age ≥ 75 years old [3–10]. Depression is defined as a mood disorder that is accompanied by persistent sadness feeling and having no interest in previously interesting activities [11]. The importance of this condition is highlighted by the fact that depression remains underdiagnosed in a high percentage of the older population by physicians [12]. This draws attention to identifying risk factors and, consequently, preventing this disease by controlling them.

Several studies have introduced poor diet, low physical activity, and obesity as risk factors for depression and concluded that there are mutual social and financial contributing factors between depression, obesity, and metabolic syndrome (MetS) [13–18]. MetS is defined as a collection of central obesity, glucose intolerance, dyslipidemia, and hypertension and is one of the main risk factors for diabetes and cardiovascular disease [19].

Several studies have depicted a bidirectional association between depression and MetS. A meta-analysis of 399,494 patients demonstrated an odds ratio of 1.48% of MetS in depressed patients [20]. A cohort study of 4,446 older citizens demonstrated a 73% increased risk of depression in older adults with MetS compared to older adults without MetS [21], supported by further investigation [22]. However, this was contradicted by the findings of a cross-sectional study, where they did not show a significant association between MetS and depression in 4579 Chinese older adults [23], in line with a similar investigation in Iranian older adults [24]. Furthermore, there are inconsistencies between the observations on the association of sex, age, and components of MetS and depression [21–25]. Notably, in a meta-analysis, Moradi et al. showed a decreasing trend in the association of MetS and depression with age [20].

Only a few studies have examined the association between obesity and depression, specifically in older people. A cohort study with a ten-year follow-up period in Japanese older adults showed a negative relation between obesity and the severity of depressive symptoms [26]. However, another study on more than nine million Chinese older adults demonstrates a significant positive association [27]. Furthermore, this study delved deeper into how the presence of metabolic syndrome components influenced this relation. Noteworthy, race and ethnicity influence the strength of the association between depression and obesity [20].

Obese individuals exhibit varying metabolic characteristics, ranging from metabolic health to MetS. Metabolic obesity phenotypes are defined based on the co-occurrence of normal or obese BMI and the presence of at least three components of MetS [28]. Due to limited research and inconsistent findings about the association between obesity phenotypes and depression, this study aims to explore this association in Iranian older adults.

## Material and methods

### Study population and sampling

This was a community-based retrospective cross-sectional study. The present investigation is a part of the Birjand Longitudinal Aging Study (**BLAS**). It was conducted on a community-dwelling ≥ 60 years old population who were residents in Birjand County, a provincial capital city in the East of Iran [29]. The sample size of this study was 1344 people.

### Eligibility criteria

The participants enrolled in this study were ≥ 60 who could communicate. Subjects who 1) were completely bedridden, 2) had very severe cognitive impairment and Alzheimer’s disease, 3) were unable to communicate, 4) were critically ill and had a very short life expectancy (less than six months), and 5) those with incomplete information were excluded.

### Sociodemographic assessment

The primary characteristics of the participants, including age, marital status, educational level, weight, height, BMI, blood pressure, waist circumference (WC), previous medical history, history of drug use and smoking, lipid profiles, liver function tests, and fasting blood sugar (FBS), were collected. Anthropometric data gathering and laboratory tests were described in the BLAS protocol study [29]. A digital scale (SECA, Germany) was used to measure the participants’ weight without shoes, which was accurate at 100 g. Heights were measured to the nearest 0.1 cm in a standing position. Body mass index (BMI) was calculated as body weight in kilograms divided by the square of height in meters. According to the World Health Organization (WHO) weight categories, participants were classified as underweight (BMI < 18.5 kg/m^2^), normal weight (18.5 ≤ BMI ≤ 24.9 kg/m^2^), overweight (25 ≤ BMI ≤ 29.9 kg/m^2^), and obese (BMI ≥ 30 kg/m^2^) [30]. Waist circumference was measured in centimeters with nonelastic tape to the nearest 0.1 mm as the distance between the smallest area under the lowest rib and above the iliac crest without pressure. Their blood pressure was measured twice using a mercury manometer while they were sitting, and the average of these two measurements was recorded.

According to the updated Adult Treatment Panel III, MetS diagnosis was made when three or more of the following criteria were present [19]:

- A WC of more than 102 cm in men or more than 88 cm in women
- Fasting serum triglyceride (TG) level of 150 mg/dl or higher
- Systolic or diastolic blood pressure levels of 130 and 85 mmHg or higher, respectively
- High-density Lipid-C (HDL) levels less than 40 mg/dl in men or less than 50 mg/dl in women
- FBS > 100 mg/dl

The obesity phenotypes were categorized as follows [28]: 1) Metabolically nonhealthy obese (MNHO), 2) Metabolically healthy obese (MHO), 3) Metabolically healthy nonobese (MHNO), and 4) Metabolically nonhealthy nonobese (MNHNO).

### Lifestyle assessment

During the interview, participants were asked about their current and past cigarette smoking and history of packs per year. Physical activity was assessed using the Longitudinal Ageing Study Amsterdam Physical Activity Questionnaire (LAPAQ), which evaluates older adults' physical activity at work and leisure time [31].

### Mood assessment

By using Patient Health Questionnaire-9 (PHQ-9), depressed mood and severity of depression were assessed. It has ten questions, including the Diagnostic and Statistical Manual of Mental Disorders (DSM-IV) depression diagnosis criteria as well as the leading symptoms of depression, scoring range from 0–27, and the last question is about the impact of difficulties that the participants experience on their daily lives. The severity of the depression is determined as: 1) none or minimal depression (Scores 0–4), 2) mild depression (Scores 5–9), 3) moderate depression (Scores 10–14), 4) moderately severe depression (Scores 15–19), and 5) severe depression (Scores ≥ 20) [32].

The presence of depressed mood and its severity were diagnosed by the score of PHQ-9 and subjects with PHQ-9 scores ≥ ten were considered to have depressed mood. [33].

### Sleep and nutrition evaluation

Duration, pattern, and sleep quality were assessed using seven selected questions of the Pittsburg Sleep Quality Index (PSQI). Furthermore, the participants were asked if they took regular naps and, if so, what the duration was. The Mini Nutritional Assessment (MNA) was used to assess nutritional status. The MNA has 16 items for screening and evaluating malnutrition in older people [34, 35]. The results divide the nutritional status into 1) adequate nutritional status, 2) protein-calorie malnutrition, and 3) at risk of malnutrition.

### Statistical analysis

Parametric continuous variables were presented by mean ± standard deviation (SD). Frequencies were shown as numbers and percentages. A t-test was used to compare the means between the two given groups. Univariate logistic regression was used to assess the odds ratio (OR) of depression in the presence of different factors. A P-value of less than 0.1 was used to enter the variables into the next model. Finally, a backward multinomial logistic regression model was used to evaluate the association between different degrees of depression and the evaluated factors with relative risk ratio (RRR). We also analyzed both genders separately to assess the effect of this modifier variable. P-values < 0.05 were considered statistically significant.

## Results

### Basic characteristics of participants

In this study, 1344 participants were enrolled, with a mean age of 69.75 ± 7.53 years. Among the participants, 268 (19.94%) had depression, and 191 (71.27%) were female. Of them, 179 (13.32%), 67 (4.99%), and 22 (1.64%) had moderate, moderate-severe, and severe symptoms, respectively. The prevalence of abdominal obesity was 52.83% (N = 710). The prevalence of obesity was 285 (21.20%). Among the obese participants, 58 (20.35%) had depression. Additionally, 78.59% of obese participants had metabolic syndrome. Of 224 participants with MNHO phenotype, 21.4% (N = 48) had depression. The prevalence of depression in MHNO, MHO, and MNHNO phenotypes was 20.2% (N = 123/608), 16.3% (N = 10/61), and 19.2% (N = 87/451), respectively.

Of 15 participants with malnourishment, 10 (75%) had depression. For the group at risk of malnourishment, 114 of 343 (33%) had depression. However, in the normal group (986), 144 had depression (11%). Furthermore, 605 (45.01%) of participants were illiterate. For a detailed summary of participant characteristics, refer to Table 1**.**

**Table 1:** Baseline characteristics of the participants

Variables		Overall N (%)	Depression status		P value
			Depressed N (%)	Non-Depressed N (%)	
Participants		1,344	268(19.94%)	1,076(80%)	
Female		696(51.79%)	191(71.27%)	505(46.93%)	< 0.001
Male		648(48.21%)	77(28.73%)	571(53.07%)	
Age (years)	60–69	753(56.03%)	136(50.75%)	617(57.34%)	0.055
	70–79	422(31.4%)	88(32.84%)	334(31.04%)	
	≥ 80	169(12.57%)	44(16.42%)	125(11.62%)	
Marital status	Yes	1,098(81.76%)	196(73.13%)	902(83.91%)	< 0.001
	No	245(18.24%)	72(26.87%)	173(16.09%)	
Live with Somebody	Yes	1,183(88.02%)	222(82.84%)	961(89.31%)	0.003
	No	161(11.98%)	46(17.16%)	115(10.69%)	
Educational level	Illiterate	605(45.01%)	170(63.43%)	435(40.43%)	< 0.001
	lower than diploma	500(37.2%)	74(27.61%)	426(39.59%)	
	Diploma	133(9.9%)	14(5.22%)	119(11.06%)	
	Academic	106(7.89%)	10(3.73%)	96(8.92%)	
Sleep duration (Hours/Day)	6–8	709(52.75%)	112(41.79%)	597(55.48%)	< 0.001
	< 6	569(42.34%)	139(51.87%)	430(39.96%)	
	> 8	66(4.91%)	17(6.34%)	49(4.55%)	
MNA total	normal	986(73.36%)	144(53.73%)	842(78.25)	< 0.001
	At risk of malnutrition	343(25.52%)	114(42.54%)	229(21.28%)	
	Malnutrition	15(1.12%)	10(3.73%)	5(0.46%)	
Current smoker	Yes	97(7.22%)	22(8.21%)	75(6.97%)	0.483
	No	1,247(92.78%)	246(91.79%)	1,001(93.03%)	
Past smoker	Yes	141(10.49%)	34(12.69%)	107(9.94%)	0.190
	No	1,203(89.51%)	234(87.31%)	969(90.06%)	
Drug	No polypharmacy	1,137(84.6%)	213(79.48%)	924(85.87%)	0.009
	Polypharmacy	207(15.4%)	55(20.52%)	152(14.13%)	
HTN	Yes	759(56.47%)	144(53.73%)	615(57.16%)	0.312
	No	585(43.53%)	124(46.27%)	461(42.84%)	
Obesity phenotypes	MHNO	608(45.24%)	123(45.9%)	485(45.07%)	0.815
	MHO	61(4.54%)	10(3.73%)	51(4.74%)	
	MNHNO	451(33.56%)	87(32.46%)	364(33.83%)	
	MNHO	224(16.67%)	48(17.91%)	176(16.36%)	
BMI (kg/m2) Categories	underweight (BMI < 18.5)	63 (4.96%)	17 (6.34%)	46 (4.28%)	0.186
	normal (18.5 ≤ BMI ≤ 24.9	484 (36.1%)	104 (38.81%)	380(35.32%)	
	overweight (25 ≤ BMI ≤ 29.9)	512 (38.10%)	89 (33.21%)	423 (39.31%)	
	obese (BMI ≥ 30)	285 (21.21%)	58 (21.64%)	227 (21.10%)	

*BMI* Body mass index, *CI* Confidence interval, *MHO* Metabolic healthy obese, *MHNO* Metabolic healthy non-obese, *MNA* Mini Nutritional Assessment, *MNHO* Metabolic non-healthy obese, *MNHNO* Metabolic non-healthy non-obese, *OR* Odds ratio, *PHQ-9* Patient Health Questionnaire-9, *PSQI* Pittsburgh Sleep Quality Index, *RRR* Relative risk ratio, *SD* Standard deviation

According to the crude analysis of the data obtained from the mentioned population, several factors showed significant differences in the primary characteristics. Female participants were significantly more than men (51.79% vs. 48.21%, P-value < 0.01). Married participants also had a significantly higher population than single ones (81.76% vs. 18.24, P-value < 0.01). With the same trend, participants with lower literacy, insufficient sleep (< 6 h/day), those who were not at risk of malnutrition, and patients without polypharmacy (taking more than five tablets per day) were significantly more than the group of patients whey were compared with (P-value < 0.01).

The results of the univariate logistic regression analysis in male participants, as displayed in Table 2**,** indicate that depression status is positively correlated with several factors. Based on this result, being in the previously smoker group (OR = 1.94, P-value:0.02, 95% CI 1.07–3.52), being at risk of malnutrition (OR = 1.91, P-value:0.03, 95% CI 1.05–3.47), or malnourished (OR = 9.68, P-value:0.03, 95% CI 1.16–80.65), and is categorized as patients with polypharmacy (OR = 4.07, P-value < 0.01, 95% CI 1.78–9.30) were associated with increased risk of depression. Mild cognitive disorder (OR = 2.58, P-value:0.01, 95% CI 1.25–5.33) and dementia (OR = 6.12, P-value < 0.01, 95% CI 2.44–15.36) were shown to be correlated with the elevated chance of depression as well. In contrast, participants in the third wealth quantile showed a statistically significant lower likelihood of depression (OR = 0.28, P-value < 0.01, 95% CI 0.11–0.73). Other factors did not significantly affect depression risk in this analysis model.

**Table 2:** Univariate logistic regression analysis in male participants

Variable	Category	Odds Ratio	P-value	95% CI
Age (years)	< 70	Reference group		
	70–79	0.99	0.99	0.54–1.84
	≥ 80	1.00	0.99	0.46–2.17
Education	Illiterate	Reference group		
	Lower than Diploma	1.55	0.20	0.79–3.06
	Diploma	2.77	0.07	0.88–8.67
	Academic	2.16	0.24	0.58–7.97
Marital status	Single	Reference group		
	Married	1.04	0.90	0.48–2.26
Currently smoker	Positive/Negative	1.94	0.05	0.98–3.84
Previously smoker	Positive/Negative	1.94	0.02	1.07–3.52
Daily sleep time (Hours)	6–8	Reference group		
	< 6	1.37	0.27	0.77–2.34
	> 8	1.01	0.97	0.34–3.01
Physical activity (Per week)	None	Reference group		
	Once	1.44	0.52	0.45–4.54
	Twice	1.36	0.49	0.55–3.31
	Three times	0.65	0.31	0.29–1.49
	≥ Five times	0.69	0.32	0.33–1.44
Malnutrition	Normal	Reference group		
	At risk of malnutrition	1.91	0.03	1.05–3.47
	Malnourished	9.68	0.03	1.16–80.65
Polypharmacy ≥ 5 tablets per day	Positive/Negative	4.07	< 0.01	1.78–9.30
Hypertension	Positive/Negative	0.75	0.38	0.39–1.41
High WC (ATP 3)	Positive/Negative	1.27	0.59	0.52–3.09
CVD history	Positive/Negative	0.86	0.63	0.46–1.59
CVA	Positive/Negative	1.45	0.44	0.55–3.81
Final DM	Positive/Negative	0.60	0.27	0.19–1.30
Wealth Quintile	Quintile 1	Reference group		
	Quintile 2	0.67	0.32	0.31–1.46
	Quintile 3	0.28	< 0.01	0.11–0.73
	Quintile 4	0.46	0.10	0.18–1.18
	Quintile 5	0.52	0.20	0.19–1.43
Cognition based on the 6-CIT criteria	None	Reference group		
	Mild Cognitive Disorder	2.58	0.01	1.25–5.33
	Dementia	6.12	< 0.01	2.44–15.36
BMI	≥ 25/ < 25	0.96	0.44	0.89–1.05

*BMI* Body mass index, *CI* Confidence interval, *MHO* Metabolic healthy obese, *MHNO* Metabolic healthy non-obese, *MNA* Mini Nutritional Assessment, *MNHO* Metabolic non-healthy obese, *MNHNO* Metabolic non-healthy non-obese, *OR* Odds ratio, *PHQ-9* Patient Health Questionnaire-9, *PSQI* Pittsburgh Sleep Quality Index, *RRR* Relative risk ratio, *SD* Standard deviation

The results of the univariate regression analysis in female participants **(**Table 3**)** demonstrate that the likelihood of depression is directly correlated with daily sleep hours of less than 6 h (OR = 1.69, P-value: < 0.01, 95%, CI 1.14–2.50), being at risk of malnutrition (OR = 2.10, P-value: < 0.01, 95% CI 1.42–3.10), or malnourished (OR = 5.69, P-value:0.02, 95% CI 1.30–24.78). In this study, the history of cardiovascular disease (OR = 1.61, P-value: 0.01, 95% CI 1.08–2.39) and dementia (OR = 1.99, P-value: 0.03, 95% CI 1.06–3.75), also revealed a positive association with the higher chance of depression. However, having high blood pressure (OR = 0.64, P-value:0.04, 95% CI 0.42–0.98) showed a statistically significant association with a lower chance of depression.

**Table 3:** Univariate logistic regression analysis in female participants

Variable	Category	Odds Ratio	P-value	95% CI^*^
Age (years)	< 70	Reference Group		
	70–79	0.95	0.83	0.61–1.48
	≥ 80	1.28	0.46	0.65–2.51
Education	Illiterate	Reference Group		
	Lower than Diploma	0.64	0.09	0.38–1.08
	Diploma	0.58	0.32	0.18–1.74
	Academic	0.74	0.63	0.22–2.48
Marital status	Single	Reference Group		
	Married	0.92	0.54	0.70–1.20
Currently smoker	Positive/Negative	1.66	0.51	0.35–7.71
Previously smoker	Positive/Negative	1.74	0.30	0.60–5.05
Daily sleep time (Hours)	6–8	Reference Group		
	< 6	1.69	< 0.01	1.14–2.50
	> 8	1.13	0.77	0.48–2.64
Physical activity (Per week)	None	Reference Group		
	Once	0.84	0.67	0.39–1.81
	Twice	0.83	0.53	0.46–1.49
	Three times	0.55	0.06	0.29–1.03
	≥ Five times	0.63	0.16	0.32–1.21
Malnutrition	Normal	Reference Group		
	At risk of malnutrition	2.10	< 0.01	1.42–3.10
	Malnourished	5.69	0.02	1.30–24.78
Polypharmacy (≥ 5 tablets per day)	Positive/Negative	1.10	0.69	0.65–1.86
Hypertension	Positive/Negative	0.64	0.04	0.42–0.98
High WC (ATP 3)	Positive/Negative	0.96	0.90	0.56–1.65
CVD history	Positive/Negative	1.61	0.01	1.08–2.39
CVA history	Positive/Negative	1.75	0.23	0.70–4.37
Final DM	Positive/Negative	1.34	0.16	0.88–2.05
Wealth Quintile	Quintile 1	Reference Group		
	Quintile 2	0.94	0.84	0.55–1.62
	Quintile 3	0.98	0.97	0.55–1.75
	Quintile 4	1.04	0.89	0.55–1.95
	Quintile 5	0.77	0.52	0.35–1.69
Cognition based on the 6-CIT Criteria	None	Reference Group		
	Mild Cognitive Disorder	1.11	0.72	0.61–2.01
	Dementia	1.99	0.03	1.06–3.75
BMI	≥ 25/ < 25	0.28	0.05	0.07–1.04

*BMI* Body mass index, *CI* Confidence interval, *MHO* Metabolic healthy obese, *MHNO* Metabolic healthy non-obese, *MNA* Mini Nutritional Assessment, *MNHO* Metabolic non-healthy obese, *MNHNO* Metabolic non-healthy non-obese, *OR* Odds ratio, *PHQ-9* Patient Health Questionnaire-9, *PSQI* Pittsburgh Sleep Quality Index, *RRR* Relative risk ratio, *SD* Standard deviation

In the results of the final model of multinomial logistic regression in female participants (Table 4), being at risk of malnutrition (RRR = 48.75, P-value:0.04, 95% CI 1.15–1980.02), and malnutrition (RRR = 3140, P-value:0.01, 95% CI 3.98–2,472,771), showed the strongest association with severe depression. Furthermore, the history of cerebrovascular accident (CVA) (RRR = 6.62, P-value < 0.01, 95% CI 1.73–25.19) and dementia (RRR = 9.73, P-value < 0.01, 95% CI 1.78–53.14), were two factors directly related to a higher risk of moderate to severe depression. The study also identifies a positive relation between being a smoker (RRR = 2.82, P-value:0.01, 95% CI 1.28–6.21), or having the previous history of cigarette smoking (RRR = 2.17, P-value:0.03, 95% CI 1.05–4.44), mild cognitive disorder (RRR = 3.40, P-value < 0.01, 95% CI 1.42–8.12), dementia (RRR = 9.63, P-value < 0.01, 95% CI 3.14–29.56), and polypharmacy (RRR = 5.21, P-value < 0.01, 95% CI 2.19–12.39), with the increased risk of moderate depression.

**Table 4:** Multiple multinomial logistic regression of depression and related factors in female participants

Variable	Category	RRR^*^	P-Value	95% CI^**^
Normal mood is the best outcome				
Mild depressive symptoms				
Age (years)	< 70	Reference group		
	70–79	0.67	0.11	0.41–1.09
	≥ 80	0.59	0.14	0.30–1.18
Education	Illiterate	Reference group		
	Lower than Diploma	1.37	0.29	0.73–2.23
	Diploma	1.57	0.30	0.66–3.74
	Academic	0.77	0.61	0.27–2.12
Marital status	Single	Reference group		
	Married	0.81	0.57	0.40–1.64
	With somebody	0.90	0.90	0.15–5.32
Currently smoker	Positive/Negative	1.16	0.63	0.62–2.15
Previously smoker	Positive/Negative	1.63	0.06	0.97–2.75
Daily sleep time	6–8	Reference group		
	< 6 h	1.16	0.49	0.74–1.82
	> 8 h	1.41	0.49	0.53–3.75
Physical activity (Per week)	None			
	Once	0.58	0.33	0.20–1.72
	Twice	0.78	0.53	0.35–1.70
	Three times	1.02	0.93	0.56–1.87
	≥ Five times	1.05	0.84	0.61–1.80
Malnutrition	Normal	Reference group		
	At risk of malnutrition	1.86	0.01	1.10–3.12
	Malnourished	4.16	0.32	0.23–73.15
Polypharmacy	Positive/Negative	0.78	0.50	0.39–1.59
Obesity phenotypes	MHNO	Reference group		
	MHO	0.45	0.23	0.12–1.65
	MNHNO	0.64	0.11	0.36–1.12
	MNHO	1.22	0.60	0.56–2.66
CVD History	Positive/Negative	1.46	0.10	0.91–2.34
CVA History	Positive/Negative	1.46	0.10	0.91–2.34
Wealth Quintile	Quintile 1	Reference group		
	Quintile 2	0.57	0.11	0.28–1.15
	Quintile 3	0.39	0.01	0.19–0.83
	Quintile 4	0.38	0.01	0.17–0.83
	Quintile 5	0.48	0.07	0.21–1.08
Cognition based on the 6- CIT questionnaire	None	Reference group		
	Mild Cognitive Disorder	1.27	0.36	0.75–2.16
	Dementia	2.23	0.04	1.00–4.97
	Moderate Depressive Disorder			
Age (years)	< 70	Reference group		
	70–79	0.87	0.73	0.41–1.85
	≥ 80	1.13	0.78	0.44–2.87
Education	Illiterate	Reference group		
	Lower than Diploma	2.15	0.07	0.92–5.00
	Diploma	3.49	0.08	0.83–14.60
	Academic	2.96	0.16	0.63–13.91
Marital status	Single	Reference Group		
	Married	1.07	0.87	0.46–2.50
Living arrangement	Alone	Reference group		
	With somebody	0.71	0.74	0.09–5.45
Currently smoker	Positive/Negative	2.82	0.01	1.28–6.21
Previously smoker	Positive/Negative	2.17	0.03	1.05–4.44
Daily sleep time (Hours)	6–8	Reference Group		
	< 6	1.38	0.35	0.69–2.78
	> 8	1.22	0.75	0.33–4.50
Physical activity (Per week)	None	Reference group		
	Once	0.60	0.15	0.29–1.21
	Twice	1.30	0.60	0.46–3.66
	Three times	0.61	0.33	0.23–1.63
	≥ Five times	0.51	0.16	0.20–1.29
MNA status	Normal	Reference group		
	At risk of malnutrition	1.58	0.22	0.75–3.36
	Malnourished	11.14	0.11	0.55–222.55
Polypharmacy	Positive/Negative	5.21	0 < 0.01	2.19–12.39
Obesity phenotypes	MHNO	Reference group		
	MHO	0.32	0.29	0.03–2.70
	MNHNO	0.63	0.29	0.27–1.48
	MNHO	0.85	0.82	0.22–3.33
CVD History	Positive/Negative	0.72	0.40	0.33–1.55
CVA History	Positive/Negative	0.85	0.83	0.20–1.55
Wealth Quintile	Quintile 1	Reference group		
	Quintile 2	0.64	0.35	0.25–1.62
	Quintile 3	0.16	< 0.01	0.05–0.51
	Quintile 4	0.24	0.01	0.07–0.77
	Quintile 5	0.45	0.19	0.13–1.49
Cognition based on the 6-CIT criteria	None	Reference group		
	Mild Cognitive Disorder	3.40	< 0.01	1.42–8.12
	Dementia	9.63	< 0.01	3.14–29.56
	Moderate-Severe Depressive Disorder			
Age	< 70 YO	Reference group		
	70–79 YO	1.18	0.76	0.38–3.67
	≥ 80 YO	0.41	0.29	0.08–2.13
Education	Illiterate	Reference group		
	Lower than Diploma	1.04	0.94	0.29–3.69
	Diploma	2.27	0.43	0.28–18.21
	Academic	1.52	0.75	0.10–22.24
Marital status	Single	Reference group		
	Married	0.63	0.64	0.09–4.21
	With somebody	0.93	0.97	0.02–42.53
Current/ex-smoker	Positive/Negative	1.13	0.86	0.26–4.85
Previously smoker	Positive/Negative	1.81	0.32	0.55–5.94
Daily sleep time (Hours)	≥ 6 and ≤ 8	Reference group		
	< 6	1.84	0.28	0.60–5.63
	> 8	2.24	0.42	0.30–16.41
Physical activity (Per week)	None	Reference group		
	Once	1.44	0.70	0.22–9.43
	Twice	1.74	0.99	–
	Three times	0.60	0.56	0.11–3.32
	≥ Five times	0.89	0.86	0.23–3.36
Malnutrition	Normal	Reference group		
	At risk of malnutrition	3.00	0.04	1.02–8.82
	Malnourished	–	–	–
Polypharmacy (Based on ≥ 5 tablets per day)	Positive/Negative	0.68	0.65	0.12–3.74
Obesity phenotypes	MHNO	Reference group		
	MHO	0.89	0.92	0.08–9.54
	MNHNO	0.27	0.13	0.04–1.52
	MNHO	0.93	0.95	0.10–8.53
CVD history	Positive/Negative	1.63	0.40	0.51–5.11
CVA history	Positive/Negative	6.62	< 0.01	1.73–25.19
Wealth Quintile	Quintile 1	Reference group		
	Quintile 2	0.34	0.16	0.07–1.54
	Quintile 3	0.29	0.14	0.05–1.54
	Quintile 4	0.38	0.29	0.06–2.31
	Quintile 5	0.42	0.39	0.06–3.00
Cognition based on the 6-CIT criteria	None	Reference group		
	Mild Cognitive Disorder	2.73	0.17	0.63–11.88
	Dementia	9.73	< 0.01	1.78–53.14
	Severe depression			
Age (years)	< 70	Reference group		
	70–79	0.37	0.59	0.00–13.78
	≥ 80	0.95	0.98	0.01–53.03
Education	Illiterate	Reference Group		
	Lower than Diploma	0.27	0.49	0.00–10.71
	Diploma	5.25	0.48	0.04–572
	Academic	Insufficient sample size		
Marital status	Single	Reference Group		
	Married	Insufficient sample size		
	With somebody	Insufficient sample size		
Currently smoker	Positive/Negative	0.23	0.55	00.00–27.93
Previously smoker	Positive/Negative	39.26	0.03	1.24–1241.29
Daily sleep time (Hours)	≥ 6 and ≤ 8	Reference Group		
	< 6	1.34	0.84	0.06–26.20
	> 8	Insufficient sample size		
Physical activity (Per week)	None	Reference group		
	Once	Insufficient sample size		
	Twice	0.11	0.24	0.18–676.62
	Three times	3.69	0.53	0.05–238.96
	≥ Five times	1.54	0.84	0.01–122.73
Malnutrition	Normal	Reference group		
	At risk of malnutrition	48.75	0.04	1.15–1980.02
	Malnourished	3140.22	0.01	3.98–2472
Polypharmacy (≥ 5 tablets per day)	Positive/Negative	Insufficient sample size		
Obesity phenotypes	MHNO	Reference group		
	MHO	Insufficient sample size		
	MNHNO	1.20	0.92	0.02–63.17
	MNHO	Insufficient sample size		
CVD history	Positive/Negative	1.01	0.99	0.03–33.98
CAD history	Positive/Negative	Insufficient sample size		
Wealth Quintile	Quintile 1	Reference Group		
	Quintile 2	1.60	0.85	00.00–274.77
	Quintile 3	0.23	0.55	00.00–27.52
	Quintile 4	3.79	0.59	0.02–492.69
	Quintile 5	Insufficient sample size		
Cognition based on the 6-CIT Criteria	None	Reference group		
	Mild Cognitive Disorder	1.28	0.89	0.02–57.49
	Dementia	Insufficient sample size		

*BMI* Body mass index, *CI* Confidence interval, *MHO* Metabolic healthy obese, *MHNO* Metabolic healthy non-obese, *MNA* Mini Nutritional Assessment, *MNHO* Metabolic non-healthy obese, *MNHNO* Metabolic non-healthy non-obese, *OR* Odds ratio, *PHQ-9* Patient Health Questionnaire-9, *PSQI* Pittsburgh Sleep Quality Index, *RRR* Relative risk ratio, *SD* Standard deviation

In contrast, in the female population, being at the third (RRR = 0.16, P-value < 0.01, 95% CI 0.05–0.51) and fourth quintiles of wealth (RRR = 0.24, P-value:0.01, 95% CI 0.07–0.77), showed association with a lower risk of moderate depression. The same pattern was observed for mild depressive symptoms as well **(**Table 4**).** However, the wealth quartiles did not show any meaningful association with moderate-severe or severe depressive symptoms.

The final model of multinomial logistic regression results being at risk of malnutrition (RRR = 8.97, 95% CI 2.46–32.72, P-value < 0.01) and malnutrition (RRR = 64.78, 95% CI 2.94–1426.495, P-value:0.01) showed the most prominent association with severe depression in male. In addition, daily sleep hours of ≤ 6 h (RRR = 4.41, 95% CI 2.08–9.38, P-value < 0.01), physical activity of Once/ week (RRR = 3.47, 95% CI 1.13–10.68, P-value:0.03), being at risk of malnutrition (RRR = 2.93, 95% CI 1.41–6.08, P-value < 0.01), and dementia (RRR = 2.56, 95% CI 1.15–5.69, P-value:0.02), were associated with the higher risk of moderate to severe depression in male **(**Table 5**)**.

**Table 5:** Multinomial logistic regression of depression and related factors in male participants

Variable	Category	RRR^*^	P-Value	95% CI^**^
Normal mood is the best outcome				
Mild depressive symptoms				
Age (years)	< 70	Reference Group		
	70–79	1.51	0.07	0.95–2.38
	≥ 80	0.63	0.25	0.29–1.38
Education	Illiterate	Reference Group		
	Lower than Diploma	1.04	0.88	0.62–1.73
	Diploma	1.36	0.50	0.55–3.37
	Academic	0.95	0.93	0.32–2.80
Marital status	Single	Reference Group		
	Married	1.12	0.40	0.85–1.48
	With somebody	0.91	0.78	0.48–1.71
Currently smoker	Positive/Negative	0.73	0.77	0.09–5.87
Previously smoker	Positive/Negative	0.99	0.99	0.22–4.27
Daily sleep time (Hours)	6–8	Reference Group		
	< 6 h	2.06	< 0.01	1.38–3.09
	> 8 h	0.39	0.06	0.15–1.05
Physical activity (Per week)	None			
	Once	1.21	0.63	0.54–2.67
	Twice	0.68	0.20	0.37–1.23
	Three times	0.35	< 0.01	0.19–0.63
	≥ Five times	0.88	0.67	0.49–1.58
Malnutrition	Normal	Reference Group		
	At risk of malnutrition	1.32	0.21	0.84–2.07
	Malnourished	1.51	0.74	0.12–18.14
Polypharmacy (≥ 5 tablets/day)	Positive/Negative	1.01	0.95	0.60–1.70
Obesity phenotypes	MHNO	Reference Group		
	MHO	0.74	0.53	0.29–1.89
	MNHNO	0.80	0.37	0.49–1.30
	MNHO	0.87	0.65	0.50–1.53
CVD History	Positive/Negative	1.50	0.06	0.96–2.32
CVA History	Positive/Negative	1.95	0.27	0.58–6.53
Wealth quintile	Quintile 1	Reference Group		
	Quintile 2	0.61	0.11	0.34–1.12
	Quintile 3	0.60	0.11	0.32–1.12
	Quintile 4	0.75	0.40	0.38–1.46
	Quintile 5	0.59	0.39	0.25–1.70
Cognition based on the 6-CIT Criteria	None	Reference Group		
	Mild Cognitive Disorder	1.55	0.11	0.89–2.71
	Dementia	1.86	0.05	0.98–3.51
Moderate depressive disorder				
Age (Years)	< 70	Reference Group		
	70–79	1.12	0.69	0.63–1.98
	≥ 80	1.07	0.86	0.46–2.48
Education	Illiterate	Reference Group		
	Lower than Diploma	0.69	0.26	0.36–1.31
	Diploma	0.61	0.49	0.15–2.45
	Academic	0.60	0.53	0.12–2.88
Marital Status	Single	Reference Group		
	Married	1.12	0.47	0.81–1.57
	With somebody	0.95	0.89	0.45–1.99
Currently smoker	Positive/Negative	1.91	0.52	0.25–14.14
Previously smoker	Positive/Negative	0.92	0.92	0.17–4.85
Daily sleep time (Hours)	6–8	Reference Group		
	< 6	2.49	< 0.01	1.50–4.14
	> 8	0.63	0.40	0.21–1.88
Physical activity (Per week)	None	Reference Group		
	Once	0.36	0.13	0.09–1.38
	Twice	0.87	0.83	0.43–1.79
	Three times	0.36	< 0.01	0.17–0.76
	≥ Five times	0.50	0.12	0.21–1.19
Malnutrition	Normal	Reference Group		
	At risk of malnutrition	2.06	< 0.01	1.22–3.47
	Malnourished	6.27	0.11	0.63–62.17
Polypharmacy (> 5 tablets/day)	Positive/Negative	1.11	0.72	0.59–2.09
Obesity phenotypes	MHNO	Reference Group		
	MHO	0.39	0.15	0.11–1.41
	MNHNO	0.48	0.01	0.27–0.85
	MNHO	0.60	0.14	0.30–1.19
Cardiovascular disease history	Positive/Negative	2.04	< 0.01	1.21–3.43
CVA History	Positive/Negative	2.23	0.23	0.60–8.29
Wealth Quintile	Quintile 1	Reference Group		
	Quintile 2	0.66	0.26	0.32–1.35
	Quintile 3	0.90	0.79	0.43–1.88
	Quintile 4	1.04	0.90	0.46–2.35
	Quintile 5	0.75	0.58	0.28–2.02
Cognition based on the 6-CIT Criteria	None	Reference Group		
	Mild Cognitive Disorder	1.56	0.22	0.75–3.26
	Dementia	2.56	0.02	1.15–5.69
Moderate-Severe Depressive Disorder				
Age (years)	< 70	Reference Group		
	70–79	1.18	0.68	0.53–2.62
	≥ 80	0.43	0.23	0.11–1.71
Education	Illiterate	Reference Group		
	Lower than Diploma	0.35	0.07	0.11–1.11
	Diploma	0.46	0.54	0.04–5.46
	Academic	1.10	0.17	0.13–8.89
Marital status	Single	Reference Group		
	Married	0.82	0.47	0.49–1.38
Living arrangement	Alone	Reference Group		
	With somebody	0.96	0.95	0.31–3.00
Currently smoker	Positive/Negative	1.07	0.95	0.07–15.99
Previously smoker	Positive/Negative	3.25	0.18	0.57–18.40
Daily sleep time (Hours)	6–8 h	Reference Group		
	< 6 h	4.41	< 0.01	2.08–9.38
	> 8 h	0.50	0.44	0.09–2.85
Physical activity (Per week)	None	Reference Group		
	Once	3.47	0.03	1.13–10.68
	Twice	0.58	0.38	0.16–1.99
	Three times	0.38	0.11	0.11–1.25
	≥ Five times	1.34	0.59	0.45–3.98
Malnutrition status	Normal	Reference Group		
	At risk of malnutrition	2.93	< 0.01	1.41–6.08
	Malnourished	2.57	0.53	0.13–49.63
Polypharmacy	Positive/Negative	0.97	0.95	0.39–2.42
Obesity phenotypes	MHNO	Reference Group		
	MHO	0.65	0.64	0.11—3.87
	MNHNO	0.55	0.16	0.24–1.27
	MNHO	1.34	0.59	0.45–3.98
CVD History	Positive/Negative	1.91	0.08	0.91–4.03
CVA History	Positive/Negative	4.16	0.07	0.86–20.01
Wealth Quintile	Quintile 1	Reference Group		
	Quintile 2	0.56	0.25	0.21–1.50
	Quintile 3	0.46	0.18	0.15–1.41
	Quintile 4	0.57	0.33	0.18–1.58
	Quintile 5	0.25	0.09	0.04–1.27
Mental status	None	Reference Group		
	Mild Cognitive Disorder	1.21	0.75	0.35–4.22
	Dementia	3.21	0.07	0.89–11.56
Severe depression				
Age (Years)	< 70	Reference Group		
	70–79	1.26	0.73	0.33–4.82
	≥ 80	1.10	0.91	0.17–6.84
Education	Illiterate	Reference Group		
	Lower than Diploma	1.49	0.58	0.35–6.35
	Diploma	1.32	0.86	0.55–31.66
	Academic	Insufficient sample size		
Marital status	Single	Reference Group		
	Married	0.25	0.02	0.07–0.86
Living arrangement	Alone	Reference Group		
	With somebody	Insufficient sample size		
Currently smoker	Positive/Negative	4.72	0.15	0.54–40.82
Smoking cession	Positive/Negative	Insufficient sample size		
Daily sleep time (Hours)	6–8	Reference Group		
	< 6	0.80	0.74	0.22–2.94
	> 8	Insufficient sample size		
Physical activity (per week)	None	Reference Group		
	Once	0.80	0.84	0.08–7.57
	Twice	0.21	0.17	0.02–2.02
	Three times	0.26	0.22	0.02–2.32
	≥ Five times	0.41	0.43	0.04–3.76
Malnutrition	Normal	Reference Group		
	At risk of malnutrition	8.97	< 0.01	2.46–32.72
	Malnourished	64.78	0.01	2.94–1426.495
Polypharmacy (≥ 5 tablet per day)	Positive/Negative	0.87	0.86	0.18–4.03
Obesity phenotypes	MHNO	Reference Group		
	MHO	0.83	0.88	0.06–10.52
	MNHNO	0.52	0.36	0.13–2.09
	MNHO	0.79	0.78	0.15–4.11
CVD History	Positive/Negative	3.56	0.03	1.12–11.32
CAD History	Positive/Negative	Insufficient sample size		
Wealth Quintile	Quintile 1	Reference Group		
	Quintile 2	3.54	0.13	0.66–18.78
	Quintile 3	1.45	0.69	0.22–9.46
	Quintile 4	0.62	0.72	0.04–8.73
	Quintile 5	1.92	0.59	0.17–20.89
Mental status	None	Reference Group		
	Mild Cognitive Disorder	0.87	0.88	0.14–5.37
	Dementia	2.95	0.25	0.45–19.05

*BMI* Body mass index, *CI* Confidence interval, *MHO* Metabolic healthy obese, *MHNO* Metabolic healthy non-obese, *MNA* Mini Nutritional Assessment, *MNHO* Metabolic non-healthy obese, *MNHNO* Metabolic non-healthy non-obese, *OR* Odds ratio, *PHQ-9* Patient Health Questionnaire-9, *PSQI* Pittsburgh Sleep Quality Index, *RRR* Relative risk ratio, *SD* Standard deviation

Conversely, being married (RRR = 0.25, 95% CI 0.07–0.86, P-value:0.02) revealed an indirect association with the lower risk of moderate to severe and severe depression in male participants, respectively. Notably, the MNHNO phenotype was associated with a significantly lower risk of moderate depression (OR = 0.48, 95%, CI:0.27–0.85, P-value = 0.01).

## Discussion

This study showed that various severities of depressive symptoms correlate with different sociodemographic and medical risk factors among male and female senior citizens. Among phenotypes of obesity, none of them were significantly associated with a higher risk of depression. On the other hand, the MNHO phenotype demonstrated a significantly lower risk of depression, almost 33% lower than the reference sample. As mentioned earlier, the association between MetS, obesity, and depression is highly controversial, and no consensus association was concluded among studies [4, 36]. Al-Khatib et al. reviewed the environmental, genetic, and epigenetic pathways that link MetS, depression, and obesity. They concluded that depression is associated with unhealthy metabolic characteristics and obesity [4].

Mansur et al. reviewed the underlying mechanisms linking MetS, obesity, and mood disorders like major depressive disorder [37]. They discussed the "metabolic-mood syndrome" entity and refuted simple comorbidity, where the diseases co-occur by chance. They presented numerous pieces of evidence to explore the bidirectional relation between obesity and metabolic characteristics and depression. Several hypotheses were mentioned to justify the association between depression and obesity, including polygenetic inheritance and similar genes involved in both diseases, especially in those with early-onset depression and obesity [38], mutual endocrine pathways such as adipokines [39], inflammatory pathways, like the hypothalamic–pituitary–adrenal axis and glucocorticoids [40], developmental factors like the intrauterine environment [37], and common environmental factors like lower socioeconomic status and education self-esteem, childhood trauma, and physical activity [41, 42]. Similarities in brain structure, chemical profile, and connectivity have been explored in depression and obesity [37]. The authors accurately mention the inconsistencies between study findings and declare that not all observations support the aforementioned hypotheses [37].

We observed a meaningful decreased risk of moderate-severe depression in the MNHNO group. The MNHNO phenotype showed the lowest risk of depression across both genders for different severities of depression, followed by MHO. In contrast with our findings, Crisp et al. showed that obesity reduced the risk of depression in men [43]; however, their observations were not supported by further research [4]. Understandably, in the 1970s, the definition of obesity differed from what we use now. Methodological shortcomings, like not exploring the typical and atypical presentations of depression, can lead to heterogeneous samples [37]. Also, we should mention that the WHO classification of BMI has been proposed for all age groups. However, there is evidence that the optimal BMI for older adults differs from that of the younger population. A study suggested an ideal BMI of 31 to 32 for females and 27 to 28 for male elderlies. This was correlated with lower gait and balance problems, decreased risk of malnutrition, and higher muscle strength [44].

Older age by itself is considered an independent risk factor for depression by several studies [45–47]; however, multiple studies did not find a meaningful association between advanced age and the risk of depression [48–50]. Increasing the occurrence of several risk factors of depression, such as chronic diseases, pain experience, disability, loneliness, financial difficulties, and inevitable restriction in routine physical activity in the older age group, seems to be a reasonable etiology for increased risk of depression in elderlies [45]. However, the present study showed no significant association between age groups and the prevalence of depression. It might be because some of the known risk factors for depression have a more significant role in age that their occurrence is less expected and less effective in the older population [51]. Moreover, the sample population's characteristics, ethnicity, culture, religious beliefs, and the study method might cause this difference.

Another controversial risk factor for depression is gender. In a systematic review of 17 studies, 10 did not find gender a significant contributor to depression in the older adults, while 7 found female gender a risk factor [3]. Another systematic review of 69 studies revealed a significantly higher prevalence of depression in female older adults compared to males [52]. This conclusion is consistent with the present study's result, which showed a meaningful association between being female and the risk of depression. The main reasons for this disparity include 1) a higher number of older adults women living independently or being widowed [53], 2) older women are at a higher risk of physical illnesses and painful chronic diseases [54], 3) females in these age groups exhibit a higher prevalence of cognitive decline [55].

Educational level lower than academic level was another factor that did not show a significant association with depression. In our study, the findings regarding the correlation between lower levels of education and depression are not consistent with several studies. Tani et al., in a study of Japanese older than 65 years, showed that a low level of education correlates with a higher risk of depression [56]. Similarly, Bjelland et al., in a longitudinal study, showed that higher educational levels had a protective effect against both anxiety and depression, and this effect was more prominent in the older population [57]. This association might be attributed to the effect of higher education on improving socioeconomic and financial status. Furthermore, several studies have identified a low socioeconomic class as a risk factor [50, 58, 59] for depression. These results were confirmed by studies on the European [47] and Taiwanese population [60].

Being a past smoker (quitting more than six months ago) in contrast with being a smoker was significantly associated with depression in the present study results. The association between depression and being an ex-smoker is consistent with the English longitudinal aging study results, which revealed the same conclusion, but they have shown that being a smoker is connected to depression, which was in contrast with our findings. This difference might be due to the low number of smokers in this study (7% of the population). However, other factors such as demographic characteristics and the overall pack-year of cigarettes these participants smoked were attributed to this difference. They also demonstrated that although smoke cessation has beneficial effects on mental health, past smokers are still at a higher risk of depression compared to non-smokers [61]. Several illustrations are suggested for this association, such as mutual genetic, environmental, and psychological factors among these two, the higher risk of smoking among those with psychological issues, and also the effect of smoking on mental health [62–64]. Sleep duration of less than six hours was another remarkably correlated factor with depression among the present study’s participants. Aligned with this result, Sun et al. revealed an association between short sleep duration and a higher risk of depression [65]. It has been shown that psychological and behavioral interventions that increase sleep duration in the sleep-deprived population would also improve mood symptoms [66].

In the current study, physical activity with an average of three times per week showed a strong association with a lower risk of depression. Similarly, Dong et al. evaluated the effect of physical activity on depression risk and found it to be a remarkable protective factor [58]. This protective effect might be caused by reducing stress levels [67]. Mumba et al. demonstrated a direct association between a sedentary lifestyle and a higher risk of depression [68, 69].

Furthermore, we observed that malnutrition had an association with depression in the present study. These two conditions have shown a bidirectional impact on each other [70]. Alam et al. and Cabrera et al. showed a similar trend and demonstrated that depressive disorders can considerably affect the appetite and may cause malnutrition [71], and inversely, lack of sufficient intake of nutrients and calories due to difficulty in chewing, swallowing, or atrophy of gastrointestinal organs might be a contributor to the severity of depression [72]. Polypharmacy showed a significant association with the risk of depression among this study’s evaluated population. It is consistent with several other studies that showed that taking multiple medications would be an independent risk factor for depression, such as Palapinyo S. et al., who revealed that there is a direct association between the number of medications and the risk of depression [73].

This study had several limitations. First, we inherently only investigated the association between depression, obesity phenotypes, and other related factors, not causal relations. We did not evaluate the various depressive presentations of major depressive disorders, which consist of typical and atypical types with decreased and increased appetite.

## Conclusion

Depression is a common debilitating mental health disorder among older adults people. Evaluating the associated factors, the present study showed a significant association between a higher risk of depression and lower educational level, current or previous smoking habits, sleep less than six hours per day, polypharmacy, and malnutrition. In contrast, physical activity three times per week and nonobese patients with metabolic syndrome revealed a remarkably lower risk of incidence of depression. Moreover, we could not demonstrate a strong relation between obesity phenotypes and the severity of depressive symptoms. However, various cohort and cross-sectional studies have had different results. It seems more investigations are needed to identify significant risk factors of depression among senior citizens and tackle the increasing prevalence of depression in this age group by controlling the risk factors.

## Data Availability

Further data will be available by the corresponding author upon reasonable request.
